# Seroprevalence of *Anaplasma phagocytophilum* Antibodies Following Tick Bites: A Serosurvey in a Tertiary Care Hospital in Romania

**DOI:** 10.3390/microorganisms13081758

**Published:** 2025-07-28

**Authors:** Cristina Alexandra Cheran, Diana Gabriela Iacob, Georgiana Neagu, Andreea Madalina Panciu, Adriana Hristea

**Affiliations:** 1 Faculty of Medicine, Carol Davila University of Medicine and Pharmacy, 020021 Bucharest, Romania; dianagiacob@gmail.com (D.G.I.); andreea-madalina.firtat@drd.umfcd.ro (A.M.P.); adriana.hristea@umfcd.ro (A.H.); 2 Department of Infectious Diseases, Bucharest Emergency University Hospital, 050098 Bucharest, Romania; 3 “Prof. Dr. Matei Bals” National Institute of Infectious Diseases, 021105 Bucharest, Romania; georgiana.neagu@rez.umfcd.ro

**Keywords:** *Anaplasma phagocytophilum*, human granulocytic anaplasmosis, anaplasmosis, serosurvey, tick bite, tick-borne infections, Romania

## Abstract

Human granulocytic anaplasmosis is an emerging tick-borne disease. Although *Anaplasma phagocytophilum* has been identified in vectors and animal reservoirs in Romania, evidence of human exposure has not yet been reported. This study aimed to generate initial evidence of human infection by evaluating *A. phagocytophilum* antibodies in individuals with recent tick exposure. We conducted a cross-sectional serosurvey between 2023 and 2024 at a tertiary care hospital in Bucharest, enrolling 80 participants 4 to 12 weeks following a tick bite. Serum IgG antibodies against *A. phagocytophilum* were detected using an indirect immunofluorescence assay, with a titer of ≥1:64 considered indicative of seropositivity. Eight (10%) participants tested positive for *A. phagocytophilum* IgG antibodies. Seropositivity was not significantly associated with demographics, geographical region, or clinical symptoms. However, fatigue and myalgia were more frequently seen in *A. phagocytophilum* IgG seropositive individuals. Notably, 43.8% of all participants reported erythema migrans, including five of the eight individuals with positive *A. phagocytophilum* IgG serology. This study provides the first serological evidence of human exposure to *A. phagocytophilum* in Romania. A 10% seroprevalence in this high-risk group suggests that anaplasmosis may be underrecognized. Clinicians should consider it in patients with tick exposure and compatible symptoms.

## 1. Introduction

In Romania, a country with an important biodiversity and habitats that favour tick populations, Lyme disease is the most well-known tick-borne infection, although other diseases transmitted by ticks might be of public health interest [1]. Among these, human granulocytic anaplasmosis (HGA), caused by the Gram-negative, obligately intracellular bacterium *Anaplasma phagocytophilum*, is transmitted primarily through tick bites, with *Ixodes ricinus* and *Ixodes persulcatus* being the vectors in Europe [2,3]. Of these, *I. ricinus* is the most prevalent tick species in Romania, making it the primary vector of concern [4]. The reservoir is represented by wild ruminants, similar to other tick-borne pathogens, such as *Borrelia* spp. [5]. Co-infections with other tick-borne pathogens are also described, especially with *Borrelia* spp. and *Rickettsia* spp. [6].

HGA was initially recognized as a distinct clinical entity in the mid-1990s, though it was previously referred to as human granulocytic ehrlichiosis (HGE) due to its initial classification within the *Ehrlichia* genus [7]. While the bacterium has been identified in vectors and animals across Europe for decades [3,8], the reporting of HGA cases remains inconsistent among countries. Unlike other infectious diseases, HGA is not subject to mandatory surveillance, which limits the availability of centralized data and may contribute to an underestimation of its true prevalence.

HGA typically develops after an incubation period of 7 to 14 days post-exposure. It is usually a self-limiting, mild infection, with a non-specific clinical picture, but can lead to severe complications, including death in immunocompromised patients [6,9]. Diagnosis typically involves antibody detection through immunofluorescence assay, detection of granulocytic inclusions on peripheral blood smear, and sometimes *A. phagocytophilum* PCR (limited to reference centres) [6,10]. Isolation of *A. phagocytophilum* in cell cultures is also possible, using HL-60 promyelocytic cell lines or tick cell lines [11,12,13].

The presence of *A. phagocytophilum* in Romania has been demonstrated by DNA detection in ticks collected from humans and in the blood of various animal species, both wild and domestic, often in co-infections with *Borrelia burgdorferi* sensu lato. Specifically, PCR testing of wild carnivores between 2014 and 2018 revealed a positivity rate for *A. phagocytophilum* of 10.5% [14], while a broader study identified only 2.5% positive samples in small mammals like mice and squirrels [15].

Furthermore, a study investigating the seroprevalence of different tick-borne diseases in horses in Romania showed that 10.3% had antibodies against *A. phagocytophilum*, 18.8% against *B. burgdorferi* s.l., and 0.5% against *Ehrlichia* spp., while 3.1% had antibodies for two or more pathogens, indicating natural exposure to these pathogens transmitted by ticks [16].

In 2017, a Romanian research team investigated questing and engorged *Ixodes ricinus* ticks, as well as tissue samples from wild animals, using PCR to screen for 44 pathogens. *Anaplasma phagocytophilum* DNA was detected in 24.2% of questing ticks and 60.4% of engorged ticks. While most questing ticks carried a single pathogen, co-infections were observed in only 27.4% of cases, compared to 69.2% among engorged ticks [17].

Another multiannual study performed in Romania, evaluating the risk of exposure to *A. phagocytophilum* and *Rickettsiae* associated with *I.ricinus* tick bites, determined a mean prevalence of 5.5% *A. phagocytophilum* in ticks collected from humans [18].

Despite the established presence of *A. phagocytophilum* in Romania in vectors and animal reservoirs, human cases of anaplasmosis have not yet been documented. This study aims to address this gap by assessing the seroprevalence of *A. phagocytophilum* antibodies in a cohort of patients presenting with tick bites in a monodisciplinary tertiary infectious diseases facility in Bucharest, Romania, thereby providing the first insight into human exposure to this pathogen within our country.

## 2. Materials and Methods

### 2.1. Study Design

We conducted a cross-sectional serosurvey to evaluate *A. phagocytophilum* serum antibodies in patients who presented in our clinic after a recent tick bite (4–12 weeks interval after the tick bite). All participants had their ticks removed either by themselves at home or by medical staff at another facility; however, none of the ticks were identified.

To ensure sufficient antibody development and avoid potential interference from antibiotic prophylaxis, participants were included only if they experienced a tick bite at least four weeks prior to serology testing and they had not received doxycycline prophylaxis after the tick bite. Exclusion criteria included refusal to complete the questionnaire, lack of specific consent, and the presence of severe immunodeficiencies or treatment with immunosuppressive agents that could affect immune responses. Since the participants first visited our clinic at least a week after the tick bite and returned later for blood sample collection between 4 and 12 weeks post-bite, we were unable to determine the species of the ticks involved in each case.

To decide how many people to include in our study, we considered what proportion of the population might have antibodies against *A. phagocytophilum.* Since there was no existing data for humans in Romania, we based our estimate on similar studies in other regions and on the study performed in Cluj in ticks infected with *A. phagocytophilum* recruited from humans after tick bite [18]. The sample size was calculated based on an expected seroprevalence of 5%, with a 95% confidence level and a 5% margin of error. We used the standard formula for estimating proportions n = [Z^2^ × P × (1 − P)]/E^2^, where Z is the Z-score for a 95% confidence level, which is 1.96, P is the expected seroprevalence, which we set at 5% (0.05), and E is the margin of error, set at 5% (0.05) to balance precision with feasibility. Therefore, 73 participants were needed to achieve sufficient statistical power for the study.

### 2.2. Collection of Blood Samples and Questionnaires

Blood samples were collected by a certified nurse using SST II Advance BD 5 mL tubes, centrifuged, and stored at −20 °C until analysis. Along with the 80 blood samples collected, participants answered a questionnaire gathering demographic information (age, gender) and clinical history related to the tick bite (date, geographical area where tick bite occurred, any clinical symptoms experienced within 3 weeks after the tick bite).

### 2.3. Detection of Antibodies Against A. phagocytophilum

We determined the presence of IgG antibodies against *A. phagocytophilum* by Indirect Immunofluorescence Assay (IFA) in collaboration with an external laboratory in Germany, due to the unavailability of test kits and reagents in our country, using the DiaSorin Molecular *A. phagocytophilum* IFA IgG assay IF1450G according to the manufacturer’s recommendations (Focus Diagnostics, Cypress, CA, USA). This IFA IgG assay utilizes HGE-1 strain-infected HL60 tick cells. IgG levels often are detectable about 7 to 10 days post-infection, peaking at 14 to 21 days and persisting for approximately a year.

The test principle of the IFA assay is a two-stage “sandwich” procedure. First, patient serum is diluted in phosphate-buffered saline (PBS) and applied to a slide containing fixed antigen, and incubated. After incubation, unbound antibodies are washed away. In the second stage, fluorescein-labeled anti-human IgG is added, allowing for the visualization of antigen–antibody complexes. The slide is then washed, dried, and mounted. Fluorescent microscopy is used to detect apple-green fluorescence within morulae, indicating a positive reaction. Semi-quantitative endpoint titers are determined by serial dilution of positive samples.

Fluorescence intensity is evaluated microscopically and graded based on the visibility of apple-green fluorescence within morulae. A result graded as 2+ to 4+ indicates moderate to intense fluorescence, while 1+ denotes definite but dim fluorescence comparable to the detectable control. A negative result shows no fluorescence or only background staining equivalent to the non-detectable control.

The serum endpoint titer is defined as the highest dilution of the sample that still demonstrates definite (1+) fluorescence. Single IgG serum endpoint titers ≥1:64 are indicative of either past infection or early response to a recent infection, while titers <1:64 are interpreted as negative [19].

### 2.4. Statistical Analysis

Data were analysed using SPSS version 26 (IBM Corp., Armonk, NY, USA). Associations between seropositivity and demographic or clinical factors were evaluated using chi-square test or Fischer test. Descriptive statistics for categorical variables were presented as numbers and percentages. Continuous variables were recorded as medians (25th–75th percentiles, IQR) or means ± standard deviation (SD). A *p* value < 0.05 was considered statistically significant.

### 2.5. Ethical Committee Approval

This study was conducted in accordance with the Declaration of Helsinki and approved by the Institutional Ethics Committee of “Prof. Dr. Matei Bals” National Institute of Infectious Diseases, Bucharest, Romania (C07704—approval date: 30 June 2023; C06727—approval date: 28 June 2024).

## 3. Results

**Figure 1:**
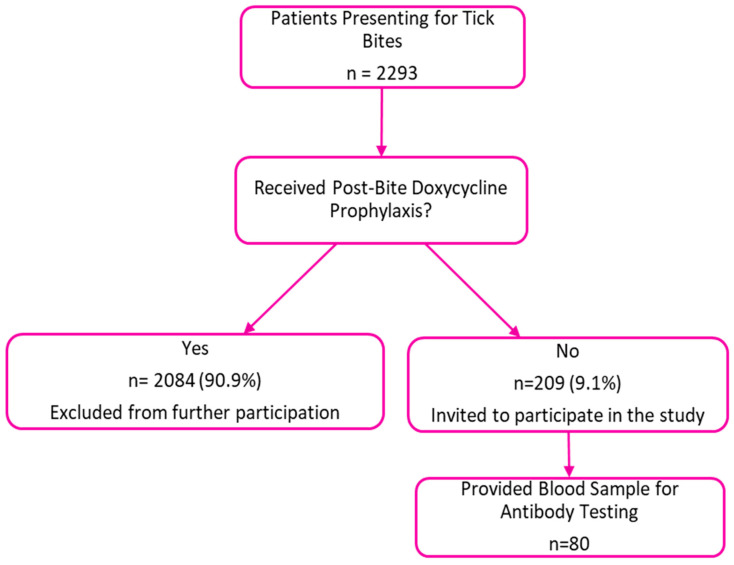
Patients presenting with tick bites during study period.

During the study period (2023–2024), 2293 patients presented for tick bites in our clinic. Among them, 2084 (90.9%) received post-bite prophylaxis with doxycycline (Figure 1). The remaining 209 patients who did not receive prophylaxis were invited to participate in our study providing a blood sample for antibody testing and 80 subjects agreed to participate.

In Table 1 are summarized the demographic characteristics, tick bite history and exposure to ticks, as well as the clinical findings after the tick bite.

Of the 80 total participants, 28 were male (35%) and 52 were female (65%). The observed seroprevalence of *A. phagocytophilum* IgG antibodies was 10%. The eight seropositive cases were evenly split between sexes, with four males and four females testing positive. Nevertheless, the proportion of seropositivity was higher among males (14.3%) than in females (7.7%), but this difference was not statistically significant (*p* = 0.4; OR 0.5 (0.1–2.1)).

The median age of participants was 46 (IQR 35–57) years, with no significant difference between individuals with or without *A. phagocytophilum* IgG antibodies.

Tick bites occurred in 48 (60%) cases in the Bucharest–Ilfov region, with the remaining 32 (40%) cases being distributed across 13 counties (Arges, Bacau, Braila, Calarasi, Dambovita, Giurgiu, Gorj, Ialomita, Olt, Prahova, Sibiu, Teleorman, Valcea), mostly in the southern part of the country. There was no difference between people with or without *A. phagocytophilum* IgG antibodies regarding past history of tick bites, activities associated with possible tick exposure, or tick bite occurring in rural areas.

In 48 cases (60%), tick bites occurred during outdoor activities such as walking or hiking in parks or forested areas. Additionally, 27 (33.8%) occurred during gardening, and three (3.8%) during fishing. Occupational exposure was noted in two (2.5%) cases, in veterinary technicians.

Of the subjects with *A. phagocytophilum* IgG-positive serology, 50% were from the Bucharest–Ilfov region, with the remaining cases distributed among Arges, Dambovita, Ialomita, and Prahova counties (12.5% each).

Regarding the anatomical region of the tick bite, 43 (53.8%) were reported on the lower limb; of these, five (6.3%) also reported multiple tick bites in other anatomical regions during the same episode.

Erythema migrans was noted in 35 (43.8%) of the participants. Among them, 17 (21.3% of the total sample) experienced erythema migrans as the only clinical manifestation after the tick bite. Interestingly, five out of eight subjects with *A. phagocytophilum* IgG-positive serology developed erythema migrans. Independently of our study, nine of the 35 subjects with erythema migrans (25.7%) were tested for IgM against *B. burgdorferi* s.l. via ELISA, at the indication of their doctor, and eight (88.8%) were positive. One of these individuals (11.1%) was also seropositive for *A. phagocytophilum* IgG in our cohort.

In the group of participants with *A. phagocytophilum* IgG-positive serology vs. negative serology there were higher percentage of fatigue (62% vs. 36%) and muscle pain (50% vs. 22%), respectively, although the difference was not statistically significant. All patients with erythema migrans received treatment with doxycycline or amoxicillin for 10–14 days.

Overall, the clinical presentation reported by the participants was non-specific. Forty-seven (58.7%) of the participants, including 18 with erythema migrans, noted multiple symptoms, while 16 (20%) participants reported no symptoms after the tick bite.

## 4. Discussion

The current serosurvey analysed samples from 80 participants with a recent history of tick bites. This represents the first serological evidence of human exposure to *A. phagocytophilum* in Romania, providing information on the presence of this disease in our country.

Seroprevalence studies in Europe indicate a widespread exposure to this bacterium in humans. A 50-year meta-analysis on *A. phagocytophilum* human infections provided an estimated overall prevalence of 8.13% (7018 cases), with 7.95% prevalence in Europe, and 11.07% in North America. The relatively low number of reported clinical cases in Europe compared to North America suggests potential underdiagnosis and/or a high proportion of paucisymptomatic infections. A higher prevalence of this infection was found among occupationally exposed patients (forestry workers, farmers, veterinarians, animal handlers, and recreational hunters) [9].

Ticks are important vectors of *A. phagocytophilum* in Europe, and several species have been documented in the southern regions of Romania. Among these, *I. ricinus* stands out as the principal vector of *A. phagocytophilum* across the continent, including Romania, due to its wide distribution and ability to parasitize a broad range of hosts such as rodents, deer, livestock, and humans. This species is frequently encountered in forested and rural habitats and has been consistently implicated in the transmission cycle of *A. phagocytophilum* to both animals and people [2,4]. In contrast, other tick species reported in southern Romania, such as *Haemaphysalis punctata* and *Dermacentor marginatus*, although prevalent in livestock surveys—particularly in Prahova County, where they accounted for approximately 66.5% and 24.5% of collected specimens, respectively—are not regarded as major vectors. There is some evidence that these species may occasionally harbor *A. phagocytophilum* DNA, but their epidemiological role in its transmission remains uncertain and is likely secondary or incidental [20]. The high prevalence of these species in livestock may simply reflect local ecological conditions rather than significant vector competence. Therefore, when considering the risk of *A. phagocytophilum* exposure in southern Romania, *I. ricinus* should be recognized as the dominant and most relevant vector, while other species play a lesser or poorly defined role.

While our study did not include direct entomological sampling, existing data points to a high prevalence of *I. ricinus* ticks in the Bucharest–Ilfov region and surrounding areas [4]. Regional environmental assessments and recent tick surveillance reports suggest that the tick population density is substantial, related to favourable ecological conditions such as forested landscapes, mild climate, and high biodiversity. These factors support consistent human exposure risk, particularly during outdoor activities and occupational tasks involving close contact with natural environments.

The seroprevalence of *A. phagocytophilum* antibodies of 10% observed in our study aligns with previous findings in endemic regions, falling within the range reported in other European studies performed on patients with a history of tick bite, from 7.5% in Bavaria, Germany to 25% in Slovakia [21,22]. Since the IgG serostatus for *A. phagocytophilum* was not assessed at inclusion in the study, it is not clear if the serologically documented infection occurred after the current tick bite or previously. However, only three participants in the seropositive group reported previous tick bites.

Given the known presence of the pathogen in animal reservoirs, individuals working closely with animals, such as veterinarians, farmers, forestry workers, and animal handlers, may be at increased risk of infection [9]. Despite our study identifying only two tick bites occurring in veterinary technicians (one in the seropositive group), likely due to the small representation of this high-risk group, this finding highlights the potential for occupational exposure to *A. phagocytophilum*. Further research is therefore needed to properly assess the risk of anaplasmosis across different occupationally exposed groups.

Excluding patients who received doxycycline prophylaxis that might interfere with the antibody development against *A. phagocytophilum* enhances the reliability of the seroprevalence within this specific high-risk group of subjects with tick bite history. In our centre, doxycycline prophylaxis post-tick bite, recommended by the United States-based guideline from the Infectious Diseases Society of America (IDSA) [23], is a common practice, based on the perception that Lyme disease is a considerable risk after tick bites. This practice has prevented us from enrolling more subjects. Indeed, in a study estimating the incidence of symptomatic *B. burgdorferi* s.l. infection in north-western and central Romania using a seroprevalence-based approach, the authors showed that the incidence of symptomatic *B. burgdorferi* s.l. infection is notably higher than the incidence of surveillance-reported Lyme disease cases [24].

Tick bites in our study were most frequently reported in the Bucharest–Ilfov region (60%), with a slight majority in the rural areas (especially in forest areas) and the remaining cases being noted across 13 other counties. However, the distribution of *A. phagocytophilum* IgG-positive cases did not differ significantly between geographical regions. Considering the known presence of *A. phagocytophilum* in ticks and animal reservoirs throughout Romania [14,15,16,17,18], the lack of significant regional variation in seropositivity was not entirely unexpected. The widespread presence of *A. phagocytophilum* is further supported by the detection of the pathogen in questing *I. ricinus* ticks across Romania, with evidence of infection identified in 34 out of the 40 counties studied [25]. While our findings might suggest a relatively homogenous risk across the studied area, it is important to acknowledge potential selection bias, as our sample was drawn from a tertiary care centre and may not be representative of the general population; consequently, the seroprevalence might differ in other settings.

From a clinical standpoint, a substantial proportion of participants (58.7%) reported multiple symptoms following tick bites, with erythema migrans (43.8%), fatigue (38.8%), headache (31.3%), and myalgia (25%) being the most common. Symptoms such as erythema migrans (62.5% vs. 37.5%) and fatigue (62.5% vs. 36.1%) were more frequent in the *A. phagocytophilum* seropositive group compared to the seronegative group; however, these differences were not statistically significant, possibly due to the limited sample size. Given the high prevalence of these non-specific symptoms, relying solely on clinical presentation makes it challenging to differentiate anaplasmosis from other tick-borne diseases or even common viral infections. Therefore, increased awareness among clinicians regarding the possibility of tick-borne infections and the importance of laboratory testing for confirmation is needed, especially in symptomatic patients with a history of tick bite.

The high prevalence of erythema migrans complicates the diagnosis of tick-borne diseases in our region, as it often leads to a presumptive diagnosis of Lyme disease and empirical treatment with doxycycline, with the consequence of overlooking *A. phagocytophilum* infections or other co-infections. Epidemiological data supports this complexity, as a systematic review of published cases of HGA revealed that co-infections with *A. phagocytophilum* and other tick-borne pathogens occurred in 9.1% cases, with *Borrelia* spp. being the most prevalent at 6.4% [6].

Erythema migrans is primarily diagnosed based on its characteristic clinical appearance and a recent history of tick bite. According to guidelines from the IDSA and the ESCMID study group for Lyme borreliosis, doxycycline treatment is recommended without prior serology testing, as approximately 50% of patients in early Lyme disease stages remain seronegative, with antibodies typically developing after 4–6 weeks [23,26]. Therefore, only nine of the subjects with EM included in our study were also tested for *B. burgdorferi* s.l. infection; of these, one tested positive in the group also positive for *A. phagocytophilum*. Confirmatory Western blot was available only through specialized laboratories, but was not further pursued.

It is noteworthy that a substantial percentage of subjects developed erythema migrans after the tick bite, with an important number among those with the presence of IgG antibodies against *A. phagocytophilum*. The design of the study does not allow us to be certain that co-infection has occurred within these subjects. Nevertheless, this observation highlights the potential for co-infections in our region, and the complexities of diagnosing tick-borne diseases, as co-infections can modify the clinical presentation and potentially impact outcomes.

The evaluation of cross-reactivity of the *A. phagocytophilum* IFA IgG assay revealed low rates for most pathogens tested. Specifically, cross-reactivity was observed in 6.7% of tick-borne encephalitis cases, 8.0% of Mediterranean spotted fever (*Rickettsia conorii*) cases, and 10.0% of acute Q fever (*Coxiella burnetii*) cases [19]. Regarding the possibility of cross-reactivity with tick-borne encephalitis virus (TBEV) in our country, data are scarce, but the TBEV infection risk is reported mainly in Transylvania and a passive surveillance system based on reported cases was implemented in the north-western counties [27].

The specificity of the DiaSorin Molecular *A. phagocytophilum* IFA IgG assay was evaluated using 85 serum samples from unexposed blood donors, including 60 from a non-endemic (France, California) and 25 from an endemic area (Slovenia). The assay demonstrated 100% specificity, with all samples yielding negative results [19].

Sensitivity with PCR/blood smear positives by time was evaluated using 30 serum samples from 15 patients with confirmed *A. phagocytophilum* infection. Diagnosis was based on the presence of an acute febrile illness, a history of *Ixodes* tick bite in an endemic area, and laboratory confirmation by either PCR and/or blood smear (positive if intracytoplasmic morulae were identified in blood, bone marrow, or cerebrospinal fluid). Sensitivity of the DiaSorin Molecular *A. phagocytophilum* IFA IgG assay was stratified based on the time elapsed from symptom onset. Of the 30 serum samples, 14 were collected within 60 days of symptom onset, 13 beyond 60 days, and three at an unknown interval. The assay showed a sensitivity of 100% (14/14) for samples obtained within 60 days and 76.9% (10/13) for those collected after 60 days, indicating reduced sensitivity over time [19].

While our study did not identify statistically significant associations between *A. phagocytophilum* seropositivity and the examined demographic factors (age, gender, rural vs. urban tick bite location, prior tick bites, outdoor activities) or reported clinical symptoms, we found a high seroprevalence rate of 10% in a population exposed to tick bites. The absence of a statistically significant association between IgG seropositivity for *A. phagocytophilum* and reported risk factors—such as tick bites occurring in rural areas, a history of prior tick bites, and frequent outdoor activities—may be explained by several factors. First, these exposures were highly prevalent across the entire study population, resulting in minimal variability between seropositive and seronegative groups and limiting the ability of these variables to distinguish between them. Additionally, recall bias or misclassification of exposure history (for example, under- or over-reporting of tick bites) could have diluted potential associations. Moreover, IgG antibodies reflect past exposure rather than recent infection, and the timing or location of exposure may not align with the recorded behaviors. Finally, other unmeasured factors, such as differences in individual immune responses, tick species, or environmental micro-habitats, may have contributed to seropositivity and confounded the expected associations.

Information on the presence of *A. phagocytophilum* infection in humans in the Bucharest area warrants further investigation, both in clinical practice and in further studies on different populations and in different areas. An important limitation of our study was the lack of representativity across various regions, except maybe for the Bucharest–Ilfov region, from which most subjects came. Larger studies with participants from different geographical regions are needed to better understand the spatial distribution of HGA across Romania, particularly in the clinical setting, as are seroprevalence studies identifying environmental or other risk factors for infection. Expanding the geographical coverage in future studies would enhance representativeness and help capture potential regional variations in seroprevalence. Targeted prevention strategies are needed, especially in areas where a high rate of infection is demonstrated and among populations with high tick exposure.

## 5. Conclusions

This research fills a critical gap by providing the first documented evidence of human exposure to *A. phagocytophilum* in Romania, as demonstrated by the notable seroprevalence of *A. phagocytophilum* antibodies in subjects at risk for tick bites. Interestingly, one of the two veterinary technicians included in our study tested positive for *A. phagocytophilum* antibodies, highlighting the need for focused diagnostic approaches and increased vigilance among occupational groups with greater exposure to ticks. Ensuring timely and accurate diagnosis in these high-risk populations—such as veterinarians, animal handlers, hunters, farmers, forestry workers, laborers in wooded areas, and even recreational outdoor enthusiasts like hikers and campers—is essential for effective management and prevention of potential complications associated with *A. phagocytophilum* infection.

These findings might serve as a starting point for understanding the epidemiology of HGA in Romania, and empowers doctors to consider this previously overlooked disease in their differential diagnoses in patients with a history of tick bites.

## Figures and Tables

**Table 1:** Characteristics of the study group and distribution by *Anaplasma phagocytophilum* IgG seropositivity.

Characteristic	All Participants (N = 80)	*A. phagocytophilum* IgG Positive (n = 8)	*A. phagocytophilum * IgG Negative (n = 72)	OR (95% CI)	*p*-Value
Age (years) Median (IQR)	46 (35–57)	46.5 (24.7–66.7)	46 (35–57)		
<46 years	40 (50)	4 (50)	36 (50)	1 (0.2–4.3)	1
Male	28 (35)	4 (50)	24 (33.3)	0.5 (0.1–2.1)	0.4
Tick bite occurring in rural areas	43 (53.8)	4 (50)	39 (54.2)	1.2 (0.3–5.4)	1
History of prior tick bites	27 (33.8)	3 (37.5)	24 (33.3)	1.2 (0.3–5.4)	1
Frequent outdoor activities associated with possible tick exposure	53 (66.3)	5 (62.5)	48 (66.7)	0.8 (0.1–3.7)	1
**Clinical manifestations reported by the participants after the tick bite**					
Erythema migrans Rash non-erythema migrans Fever Headaches Fatigue Muscle pain Nausea/vomiting	35 (43.8)	5 (62.5)	30 (41.6)	2.3 (0.5–10.5)	0.3
	18 (22.5)	0	18 (25)	N/A	
	15 (18.8) 25 (31.3) 31 (38.8) 20 (25) 7 (8.8)	0 3 (37.5) 5 (62.5) 4 (50) 0	15 (20.8) 22 (30.5) 26 (36.1) 16 (22.2) 7 (9.7)	N/A 1.37 (0.3–6.2) 2.9 (0.6–13.3) 5.8 (1.2–27.1) N/A	0.7 0.2 0.2

## Data Availability

The original contributions presented in the study are included in the article. Further inquiries can be directed to the corresponding author.

## Abbreviations

The following abbreviations are used in this manuscript:

CI	Confidence Interval
DNA	Deoxyribonucleic Acid
ELISA	Enzyme-Linked Immunosorbent Assay
HGA	Human granulocytic anaplasmosis
HGE	Human granulocytic ehrlichiosis
IDSA	Infectious Diseases Society of America
IgG	Immunoglobulin G
IQR	Interquartile Range
N/A	Not Applicable
OR	Odds Ratio
PCR	Polymerase chain reaction
s.l.	sensu lato
SD	Standard Deviation
SPSS	Statistical Package for the Social Sciences
SST	Serum Separator Tube
